# Absence of human papillomavirus in oral cavity squamous cell carcinomas among Saudi patients

**DOI:** 10.1002/cre2.153

**Published:** 2019-01-29

**Authors:** Louay Jaber, Hanadi Fatani, Saleh F. Aldhahri

**Affiliations:** ^1^ College of Dentistry Imam Abdulrahman Bin Faisal University Saudi Arabia; ^2^ Department of Pathology King Fahad Medical City Saudi Arabia; ^3^ Faculty of Medicine King Saud University Saudi Arabia

**Keywords:** HPV, oral cavity squamous cell carcinoma, prevalence, Saudi Arabia

## Abstract

This study aimed to examine the possible association of human papillomavirus (HPV) with oral cavity squamous cell carcinomas (OCSCCs) in Saudi Arabia. Forty‐five paraffin‐embedded tumor blocks that represent different subsets of OCSCCs between 2010 and 2014 were retrieved and histologically evaluated. The presence of high‐risk HPV (16, 18, 31, and 33) was assessed by p16‐immunohistochemistry followed by DNA detection using in situ hybridization technique. Twenty‐four patients were male with the mean age of 59.3 years, and 21 patients were female with the mean age of 61.2 years. Forty‐one cases were positive for p16 immunostaining, and the remaining four cases were negative. However, none of the 45 cases showed DNA‐expression for any HPV subtypes (16, 18, 31, and 33). High‐risk HPV appears not to be involved in the etiology of OCSCCs in older Saudi patients, but further studies with cross section of a younger age group are still required.

## 1 INTRODUCTION

The estimated age‐standardized incidence rate of oral cancer is 2.7 per 100,000, and this rate is expected to increase globally because of population aging and growth (Shield et al., 2017). The description of oral cavity squamous cell carcinomas (OCSCCs) usually consists of oral cavity that includes the anterior 2/3 of the tongue (C02–C06; World Health Organization, 2016).

It is well known that tobacco and alcohol are considered risk factors for oral cancer (Tuyns et al., 1988). However, accumulating evidences consider human papillomavirus (HPV) as a new etiologic factor for different subsets of head and neck squamous cell carcinoma (HNSCCs; D'Souza et al., 2007; Gillison et al., 2000; International Agency for Research on Cancer, 2007)**.**

HPV‐associated OCSCCs seem to be a separate clinical entity. HPV‐positive cancers are likely to appear at young age, with reduced exposure to tobacco products and alcohol. Furthermore, the prognosis of HPV‐positive tumors is better than HPV‐negative tumors, which is partly attributed to enhanced sensitivity to radiation and chemotherapy (Chaturvedi, Engels, Anderson, & Gillison, 2008).

Although more than 100 different types of HPV are known (International Agency for Research on Cancer, 2007), the viral genotypes, which are often associated with leukoplakia and squamous cell carcinoma, are classified as high‐risk category such as HPV 16, 18, 31, and 33. In addition, HPV 16 genome is considered the most frequently encountered (84–90%) in HPV‐positive patients (D'Souza et al., 2007; Gillison et al., 2000).

Previous studies from different regions and countries on HPV association in OCSCCs have reported widely different estimates ranging from 0% to 100% (Castro & Bussoloti Filho, 2006; Elango et al., 2011; Kreimer, Cifford, Boyle, & Franceschi, 2005; Lopes et al., 2011).

In Saudi Arabia, no study has been previously investigated the possible association of high‐risk HPV with OCSCCs; therefore, the present study was designated to investigate this possibility using archived OCSCCs specimens from the Department of Pathology at King Fahad Medical City (KFMC) in Riyadh, Saudi Arabia.

## 2 MATERIAL AND METHODS

### 2.1 Tumor blocks

Following approval of the Ethics Committee at King Abdullah International Medical Research Center in Riyadh, Saudi Arabia (Ref. RO/326/2013, Date: 24/06/2013), a total of 88 formalin‐fixed paraffin‐embedded (FFPE) tumor blocks were retrospectively retrieved from pathology archives at KFMC between 2010 and 2014. These tumor blocks were cut into 3‐μm sections, and the availability of representative tumor tissue together with preservation of nucleic acids in the tissue was reconfirmed by an experienced pathologist certified by Saudi Council for Health Specialists. Accordingly, 45 tumor blocks were included in this study because of the following exclusion criteria:

1. The histological sections do not contain representative tumor tissue.
2. The histological sections fail to demonstrate positive staining when DNA positive control probe (PB0682) was used in place of the HPV probe (subtypes 16, 18, 31, and 33).

According to the International Classification of Disease (ICD‐10; World Health Organization, 2016), the distribution of specimens by anatomic subsite is shown in Table 1.

**Table 1:** Distribution of oral cavity squamous cell carcinomas by anatomic subsite

Squamous cell carcinomas		
Site	Subsite	Total number of specimens
Oral cavity (C02‐C06)	Tongue: (C02.0, 1 & C02.2) = 21	45
	Mucosa of upper and lower lips (C00.3, 4) = 1	
	Upper alveolus and gingiva (upper gum) (C03.0) = 4	
	Lower alveolus and gingiva (lower gum) (C03.1) = 4	
	Hard palate (C05.0) = 1	
	Soft palate (C05.1) = 1	
	Cheek mucosa (C06.0) = 11	
	Retromolar areas (C06.2) = 2	

### 2.2 P16‐immunohistochemistry

An automated immunostainer (Leica BOND‐III) was used to perform P16 immunostaining with antibody anti‐P16 (Thermo Scientific, clone 5A8A4, dilution 1∶3,000, pretreatment: Bond Epitope Retrieval Solution 1/97°C) and Bond Polymer Refine Detection on Leica BOND‐III. If staining attains at least 10% of the nucleus and cytoplasm, P16 immunostaining was considered positive; otherwise, P16 was considered negative (Rodrigo et al., 2015).

### 2.3 HPV—In situ hybridization

HPV‐DNA detection by in situ hybridization was completed using Leica HPV probe (subtypes 16, 18, 31, and 33), Catalogue No. PB0829, and Bond Polymer Refine Detection kit (DS9800) on Leica BOND‐III. Leica HPV probe was ready to be used; therefore, no further necessities to reconstitute, mix, dilute, or titrate this reagent.

DNA positive control probe (PB0682) was used in place of the HPV probe (subtypes 16, 18, 31, and 33) with a section of each patient's specimen. This was performed to check the preservation of nucleic acids in the tissue as well as accessibility of the probe to these nucleic acids. If the DNA positive control probe did not reveal positive staining, results were considered invalid, and the patient's specimen was excluded from this study. Furthermore, HPV probe (subtypes 16, 18, 31, and 33) was validated using previously confirmed 10 HPV‐positive and 10 HPV‐negative cervical cancer specimens, which were retrieved from pathology archives at KFMC.

DNA negative control (PB0731) was used in place of the HPV probe (subtypes 16, 18, 31, and 33) with a section of each patient's specimen. This was done to improve interpretation of specific versus nonspecific staining.

### 2.4 Statistical analysis

Sensitivity analysis was done by taking HPV as confirmatory test (gold standard) for the specimens suspected to be HPV positive in OCSCCs (Table 3).

## 3 RESULTS

All patients received their treatment at otolaryngology department at KFMC. Twenty‐four patients were male with the mean age of 59.3 years, and 21 patients were female with the mean age of 61.2 years. The stage of the tumors was determined according to the classification of TNM system of the International Union Against Cancer (Sobin, Gospodarwicz, & Wittekind, 2009; Table 2).

**Table 2:** Detection of P16 and HPV in oral cavity squamous cell carcinomas

Oral cavity squamous cell carcinomas				
Tumor stage	P16		HPV	
			16, 18, 31, 33	
	Positive	Negative	Positive	Negative
I	5	0	0	5
II	3	1	0	4
III	6	0	0	6
IV	27	3	0	30
Total	41	4	0	45

*Note*. TP: true positive; FP: false positive; FN: false negative; TN: true negative. Sensitivity = TP/(TP + FN) = 0%. Specificity = TN/(FP + TN) = 8.89%. Positive predictive value (PPV) = TP/(TP + FP) = 0%. Negative predictive value (NPV) = TN/(FN + TN) = 100%.

Forty‐one cases were positive for P16 immunostaining, and the remaining four cases were negative. DNA detection for HPV subtypes (16, 18, 31, and 33) by in situ hybridization demonstrates negative staining for all 45 specimens used in the present study (Table 2).

### 3.1 Statistical analysis

Sensitivity analysis, which was done by taking HPV as confirmatory test (gold standard) for the specimens suspected to be HPV positive in OCSCCs (Table 3), revealed 0% sensitivity, 8.89% specificity, 0% positive predictive values, and 100% negative predictive value (Table 3).

**Table 3:** Sensitivity analysis

P16	HPV		Total
	16, 18, 31, 33		
	Positive	Negative	
Positive	0 (TP)	41 (FP)	41
Negative	0 (FN)	4 (TN)	4
Total	0	45	45

## 4 DISCUSSION

Reports have shown that association of HPV with OCSCCs varies significantly from one geographic location to another (Table 4). This heterogeneity has been clearly demonstrated in a recent systematic review by Anantharaman et al. (2017), who reported that HPV16‐associated oral cancer is 10.6% in the United States, 6.1% in Europe, and 0% in Brazil. Similarly, another literature review in Asia Pacific (Shaikha, McMillan, & Johnson, 2015) has reported that HPV positivity in oral cancer attained 100% in Japan and Malaysia, 82.7% in Taiwan, 3.1% in Thailand, and 2.9% in Bangladesh.

**Table 4:** Prevalence of HPV positivity in oral cancer by country/region

PubMed ID	First author	Country/region	Number of oral cancer cases	Overall HPV positivity (%)	HPV genotypes
28108990	Anantharaman D. et al.	Brazil	107	0	16
23617206	Akhter M. et al.	Bangladesh	34	2.9	16, 18, 6, 11
19256774	Khovidhunkit S.O. et al.	Thailand	32	3.1	16
28108990	Anantharaman D. et al.	Europe	165	6.1	16
28108990	Anantharaman D. et al.	USA	123	10.6	16
28612284	Qatouseh L.A. et al.	Jordan	65	21.5	16,18
12190813	Chen P.C. et al.	Taiwan	29	82.7	16, 18, 6, 11
17181737	Koyama K. et al.	Japan	13	100	16, 18, 22, 38, 70
17487385	Lim K.P. et al.	Malaysia	20	100	16, 18

In the light of the above‐mentioned reports, the absence of high‐risk HPV subtypes (16, 18, 31, and 33) in OCSCCs among Saudi patients is not a unique phenomenon. It is suggested that this absence might be attributed to social and cultural prohibitions of certain sexual behavior such as oro‐genital contact. It is noteworthy to mention that similar suggestion has been previously proposed by Li et al. (2003). The possible implication of this sexual behavior in oral cancer has been supported by many reports showing strong association between sexual behavior and HNSCCs (D'Souza, Agrawal, Halpern, Bodison, & Gillison, 2009; Schwartz et al., 1998; Smith et al., 2004). Unfortunately, sexual behavior data are not available in our patients' file. However, in this regard, it is speculated that the results of our present study might be related to the sexual behavior status of older patients, but not necessarily represent the status of a younger and modern Saudi patients who possibly have different sexual behaviors. Accordingly, it is suggested that further studies with cross section of young and modern Saudi Arabian population are needed.

It is noteworthy to mention that prevalence of HPV‐associated oropharyngeal, hypopharyngeal, tonsils, and laryngeal cancers varies largely in the literature, ranging from 0% up to 75% (Duray et al., 2011; Halec et al., 2013; Isayeva, Li, Maswahu, & Brandwein‐Gensler, 2012; Kreimer et al., 2005; Li et al., 2003; Ribeiro et al., 2011; Rodrigo et al., 2015; Shaikha et al., 2015; Singhi & Westra, 2010). Therefore, it is suggested that large multicenter studies for HPV‐associated different subsets of HNSCCs are needed for better understanding of the existing heterogeneity between different geographic locations.

In the present study, p16 has been used as a predictor for HPV status because previous reports have shown that p16 overexpression is associated with HPV infection in oropharyngeal squamous cell carcinoma and malignant oral lesions (Fregonesi et al., 2003). However, our results have shown that majority of OCSCCs specimens (91%) was positive for p16 immunostaining with complete absence (100%) of HPV subtypes (16, 18, 31, and 33). This outcome is in line with a recent report by Belobrov et al. (2017) who demonstrated that p16 is rarely associated with HPV‐positive OCSCCs, and its overexpression might be related to nonviral mechanisms. In this regard, it has been suggested by Witkiewicz, Knudsen, Dicker, and Knudsen (2011) that p16 expression in the absence of HPV‐DNA detection might be due to RB signaling pathway disturbance, which is not related to HPV infection, such as in the case of lymphoma and small cell lung cancer. However, further studies are required for confirmation.

The type of biopsy specimens (FFPE blocks) or the technique (in situ hybridization) used in the present study is not likely to affect the results, because previous reports have shown that using paraffin‐embedded versus fresh frozen biopsies (Kreimer et al., 2005), or PCR versus in situ hybridization techniques (Mehanna et al., 2013), did not alter the positivity of HPV‐associated HNSCCs. However, it is suggested that further comparative studies using different types of biopsy specimens in combination with various analytical approach such as microarray, real‐time PCR, and in situ hybridization techniques are needed for confirmation.

P16 immunohistochemistry together with in situ hybridization technique is commonly used by pathologists for HPV‐DNA detection (Klingenberg et al., 2010; Kumar et al., 2008; Licitra et al., 2006). However, DNA degradation when using formalin fixation and paraffin‐embedded materials might be of concern because detection rates of larger HPV‐DNA fragments in FFPE samples have been found to be less when compared with shorter DNA segments (Baay et al., 1996); therefore, in our study, preservation of nucleic acids in the tissue as well as accessibility of the probe to these nucleic acids has been tested using DNA positive control probe (PB0682). It is noteworthy to mention that the newest HPV detection technique such as RNAscope™ HPV test might be promising (Mirghani et al., 2014), but further research still required regarding this issue.

It has been reported that the mean age of oral cancer in Saudi Arabia is 62 years with the highest burden in Jizan and Najran (Brown, Ravichandran, & Warnakulasuriya, 2006). Furthermore, a recent report from eight different regions in southern Saudi Arabia has shown that 33% of oral cancer patients were at the age group of >70 years, 44.7% between 51 and 70 years, 16% between 31 and 50 years, 4.3% between 10 and 30 years, and 2.1% of <10 years (Hesham et al., 2017). These later data showed that the burden of oral cancer among young Saudi patients is not large. However, the increased incidence of HPV infection in Saudi Arabia from 3.5% to 5.8% (Memish, Filemban, Al‐Hakeem, Hassan, & Al‐Tawfiq, 2016) suggests the need to establish a preventive educational programs regarding sexually transmitted infections.

It is noteworthy to mention that there are only few studies dealing with risk factors of head and neck cancer in Saudi Arabia, but cigarette smoking, shisha, betel nuts, khat, and smokeless tobacco such as shamma are considered common risk factors for head and neck cancer occurrence in this country (Alhazzazi & Alghamdi, 2016). The habits of chewing tobacco and khat are likely to be associated with higher rate of oral cancer (Sawair et al., 2007). Furthermore, shamma, which is frequently used among oral cancer patients in the southern province of Saudi Arabia (Allard, DeVol, & Te, 1999; Ibrahim et al., 1986; Quadri et al., 2015), was found to increase the incidence of oral cancer by 29‐fold (Quadri et al., 2015).

In the light of the above‐mentioned findings, it is suggested that prevention of oral cancer in this country should combine educational programs about sexually transmitted infections and tobacco cessation.

To our knowledge, this is the first study in Saudi Arabia, which report that high‐risk HPV (16, 18, 31, and 33) appears not to be involved in the etiology of different subsets of OCSCCs in older Saudi patients. However, due to the selected sample of the present study, further investigations using large sample size with cross section of a younger age group are still required.

## CONFLICT OF INTEREST

The authors declare no conflicts of interests.

## References

[cre2153-bib-0001] Alhazzazi, T. Y. , & Alghamdi, F. T. (2016). Head and neck cancer in Saudi Arabia: A systematic review. Asian Pacific Journal of Cancer Prevention, 17, 4043–4048. 27644659

[cre2153-bib-0002] Allard, W. F. , DeVol, E. B. , & Te, O. B. (1999). Smokeless tobacco (shamma) and oral cancer in Saudi Arabia. Community Dentistry and Oral Epidemiology, 27, 398–405. 10.1111/j.1600-0528.1999.tb02038.x 10600072

[cre2153-bib-0003] Anantharaman, D. , Abedi‐Ardekani, B. , Beachler, D. C. , Gheit, T. , Olshan, A. F. , Wisniewski, K. , … D'Souza, G. (2017). Geographic heterogeneity in the prevalence of human papillomavirus in head and neck cancer. International Journal of Cancer, 140, 1968–1975. 10.1002/ijc.30608 28108990 PMC8969079

[cre2153-bib-0004] Baay, M. F. , Quint, W. G. , Koudstaal, J. , Hollema, H. , Duk, J. M. , Burger, M. P. , … Herbrink, P. (1996). Comprehensive study of several general and type‐specific primer pairs for detection of human papillomavirus DNA by PCR in paraffin‐embedded cervical carcinomas. Journal of Clinical Microbiology, 34, 745–747. 8904451 10.1128/jcm.34.3.745-747.1996 PMC228883

[cre2153-bib-0005] Belobrov, S. , Cornall, A. M. , Young, R. J. , Koo, K. , Angel, C. , Wiesenfeld, D. , … McCullough, M. (2017). The role of human papillomavirus in p16‐positive oral cancers. Journal of Oral Pathology & Medicine, 00, 1–7. 10.1111/jop.12649 29024035

[cre2153-bib-0006] Brown, A. , Ravichandran, K. , & Warnakulasuriya, S. (2006). The unequal burden related to the risk of oral cancer in the different regions of the Kingdom of Saudi Arabia. Community Dental Health, 23, 101–106. 16800366

[cre2153-bib-0007] Castro, T. P. , & Bussoloti Filho, I. (2006). Prevalence of human papillomavirus (HPV) in oral cavity and oropharynx. Brazilian Journal of Otorhinolaryngology, 72, 272–282. 10.1016/S1808-8694(15)30068-9 16951865 PMC9445676

[cre2153-bib-0008] Chaturvedi, A. K. , Engels, E. A. , Anderson, W. F. , & Gillison, M. L. (2008). Incidence trends for human papillomavirus‐related and ‐unrelated oral squamous cell carcinomas in the United States. Journal of Clinical Oncology, 26, 612–619. 10.1200/JCO.2007.14.1713 18235120

[cre2153-bib-0009] D'Souza, G. , Agrawal, Y. , Halpern, J. , Bodison, S. , & Gillison, M. L. (2009). Oral sexual behaviors associated with prevalent oral human papillomavirus infection. The Journal of Infectious Diseases, 199, 1263–1269. 10.1086/597755 19320589 PMC4703086

[cre2153-bib-0010] D'Souza, G. , Kreimer, A. R. , Viscidi, R. , Pawlita, M. , Fakhry, C. , Koch, W. M. , … Gillison, M. (2007). Case–control study of human papillomavirus and oropharyngeal cancer. The New England Journal of Medicine, 356, 1944–1956. 10.1056/NEJMoa065497 17494927

[cre2153-bib-0011] Duray, A. , Descamps, G. , Arafa, M. , Decaestecker, C. , Remmelink, M. , Sirtaine, N. , … Saussez, S. (2011). High incidence of high‐risk HPV in benign and malignant lesion of the larynx. International Journal of Oncology, 39, 51–59. 10.3892/ijo.2011.1031 21573490

[cre2153-bib-0012] Elango, K. J. , Suresh, A. , Erode, E. M. , Subhadradevi, L. , Ravindran, H. K. , Iyer, S. K. , … Kuriakose, M. A. (2011). Role of human papilloma virus in oral tongue squamous cell carcinoma. Asian Pacific Journal of Cancer Prevention, 12, 889–896. 21790221

[cre2153-bib-0013] Fregonesi, P. A. G. , Teresa, D. B. , Duarte, R. A. , Neto, C. B. , de Oliveira, M. R. B. , & Soares, C. P. (2003). p16(INK4A) immunohistochemical overexpression in premalignant and malignant oral lesions infected with human papillomavirus. J Histochem Cytochem., 51, 1291–1297. 10.1177/002215540305101006 14500697

[cre2153-bib-0014] Gillison, M. L. , Koch, W. M. , Capone, R. B. , Spafford, M. , Westra, W. H. , Wu, L. , … Sidransky, D. (2000). Evidence for a causal association between human papillomavirus and a subset of head and neck cancers. Journal of the National Cancer Institute, 92, 709–720. 10.1093/jnci/92.9.709 10793107

[cre2153-bib-0015] Halec, G. , Holzinger, D. , Schmitt, M. , Flechtenmacher, C. , Dyckhoff, G. , Lloveras, B. , … Pawlita, M. (2013). Biological evidence for a causal role of HPV16 in a small fraction of laryngeal squamous cell carcinoma. British Journal of Cancer, 109, 172–183. 10.1038/bjc.2013.296 23778529 PMC3708587

[cre2153-bib-0016] Hesham, A. , Syed, K. B. , Jamal, B. T. , Alqahtani, A. M. A. , Alfaqih, A. A. A. , Alshehry, H. A. A. , … Mustafa, A. B. (2017). Incidence, clinical presentation, and demographic factors associated with oral cancer patients in the southern region of Saudi Arabia: A 10‐year retrospective study. J Int Oral Health., 9, 105–109.

[cre2153-bib-0017] IARC (2007). Monographs on the evaluation of carcinogenic risks to humans In Human papillomaviruses. France: World Health Organization Press.

[cre2153-bib-0018] Ibrahim, E. M. , Satti, M. B. , Al Idrissi, H. Y. , Higazi, M. M. , Magbool, G. M. , & Al Quorain, A. (1986). Oral cancer in Saudi Arabia: The role of alqat and alshammah. Cancer Detection and Prevention, 9, 215–218. 3742501

[cre2153-bib-0019] Isayeva, T. , Li, Y. , Maswahu, D. , & Brandwein‐Gensler, M. (2012). Human papillomavirus in non‐oropharyngeal head and neck cancers: A systematic literature review. Head and Neck Pathology, 6, S104–S120. 10.1007/s12105-012-0368-1 22782230 PMC3394168

[cre2153-bib-0020] Klingenberg, B. 1., Hafkamp, H.C. , Haesevoets, A. , Manni, J.J. , Slootweg, P.J. , Weissenborn, S.J. , Klussmann J.P. … Speel, E.J. (2010) p16 INK4A overexpression is frequently detected in tumour‐free tonsil tissue without association with HPV. Histopathology 56, 957–967. 10.1111/j.1365-2559.2010.03576.x 20636796

[cre2153-bib-0021] Kreimer, A. R. , Cifford, G. M. , Boyle, P. , & Franceschi, S. (2005). Human papillomavirus types in head and neck squamous cell carcinomas worldwide: A systematic review. Cancer Epidemiology, Biomarkers & Prevention, 14, 467–475. 10.1158/1055-9965.EPI-04-0551 15734974

[cre2153-bib-0022] Kumar, B. 1., Cordell, K.G. , Lee, J.S. , Worden, F.P. , Prince, M.E. , Tran, HH. , Wolf G.T. , Urba S.G. , Chepeha D.B. , Teknos T.N. , Eisbruch A. , Tsien C.I. , Taylor J.M.G. , D'Silva N.J. , Yang K. , Kurnit D.M. , Bauer J.A. , Bradford C.R. … Carey, T.E. (2008) EGFR, p16, HPV Titer, Bcl‐xL and p53, sex, and smoking as indicators of response to therapy and survival in oropharyngeal cancer. Journal of Clinical Oncology 26, 3128–3137. 10.1200/JCO.2007.12.7662 18474878 PMC2744895

[cre2153-bib-0023] Li, W. , Thompson, C. H. , Xin, D. , Cossart, Y. E. , O'Brien, C. J. , McNeil, E. B. , … Rose, B. R. (2003). Absence of human papillomavirus in tonsillar squamous cell carcinomas from Chinese patients. The American Journal of Pathology, 163, 2185–2189. 10.1016/S0002-9440(10)63576-6 14633593 PMC1892374

[cre2153-bib-0024] Licitra, L. , Perrone, F. , Bossi, P. , Suardi, S. , Mariani, L. , Artusi, R. , … Pilotti, S. (2006). High‐risk human papillomavirus affects prognosis in patients with surgically treated oropharyngeal squamous cell carcinoma. Journal of Clinical Oncology, 24, 5630–5636. 10.1200/JCO.2005.04.6136 17179101

[cre2153-bib-0025] Lopes, V. , Murray, P. , Williams, H. , Woodman, C. , Watkinson, J. , & Robinson, M. (2011). Squamous cell carcinoma of the oral cavity rarely harbours oncogenic human papillomavirus. Oral Oncology, 47, 698–701. 10.1016/j.oraloncology.2011.04.022 21689967

[cre2153-bib-0026] Mehanna, H. , Beech, T. , Nicholson, T. , El‐Hariry, I. , McConkey, C. , Paleri, V. , & Roberts, S. (2013). Prevalence of human papillomavirus in oropharyngeal and nonoropharyngeal head and neck cancer—Systematic review and meta‐analysis of trends by time and region. Head & Neck, 35, 747–755. 10.1002/hed.22015 22267298

[cre2153-bib-0027] Memish, Z. A. , Filemban, S. M. , Al‐Hakeem, R. F. , Hassan, M. H. , & Al‐Tawfiq, J. A. (2016). Sexually transmitted infections case notification rates in the Kingdom of Saudi Arabia, 2005–2012. Journal of Infection in Developing Countries, 31, 884–887. 10.3855/jidc.7020 27580336

[cre2153-bib-0028] Mirghani, H. , Amen, F. , Moreau, F. , Guigay, J. , Ferchiou, M. , Melkane, A. E. , … Lacau St Guily, J. (2014). Human papilloma virus testing in oropharyngeal squamous cell carcinoma: What the clinician should know. Oral Oncology, 50, 1–9. 10.1016/j.oraloncology.2013.10.008 24169585

[cre2153-bib-0029] Quadri, M. F. , Alharbi, F. , Bajonaid, A. M. , Moafa, I. H. , Sharwani, A. A. , & Alamir, A. H. (2015). Oral squamous cell carcinoma and associated risk factors in Jazan, Saudi Arabia: A hospital based case control study. Asian Pacific Journal of Cancer Prevention, 16, 4335–4338. 10.7314/APJCP.2015.16.10.4335 26028095

[cre2153-bib-0030] Ribeiro, K. B. , Levi, J. E. , Pawlita, M. , Koifman, S. , Matos, E. , Eluf‐Neto, J. , … Waterboer, T. (2011). Low human papillomavirus prevalence in head and neck cancer: Results from two large case–control studies in high‐incidence regions. International Journal of Epidemiology, 40, 489–502. 10.1093/ije/dyq249 21224273

[cre2153-bib-0031] Rodrigo, J. P. , Hermsen, M. A. , Fresno, M. F. , Brakenhoff, R. H. , García‐Velasco, F. , Snijders, P. J. , … García‐Pedrero, J. M. (2015). Prevalence of human papillomavirus in laryngeal and hypopharyngeal squamous cell carcinomas in northern Spain. Cancer Epidemiology, 39, 37–41. 10.1016/j.canep.2014.11.003 25468644

[cre2153-bib-0032] Sawair, F. A. , Al‐Mutwakel, A. , Al‐Eryani, K. , Al‐Surhy, A. , Maruyama, S. , Cheng, J. , … Saku, T. (2007). High relative frequency of oral squamous cell carcinoma in Yemen: Qat and tobacco chewing as its aetiological background. Int J Env Health Res., 17, 185–195. 10.1080/09603120701254813 17479382

[cre2153-bib-0033] Schwartz, S. M. , Daling, J. R. , Doody, D. R. , Wipf, G. C. , Carter, J. J. , Madeleine, M. M. , … Galloway, D. A. (1998). Oral cancer risk in relation to sexual history and evidence of human papillomavirus infection. Journal of the National Cancer Institute, 90, 1626–1636. 10.1093/jnci/90.21.1626 9811312

[cre2153-bib-0034] Shaikha, M. H. , McMillan, N. A. , & Johnson, N. W. (2015). HPV‐associated head and neck cancers in the Asia Pacific: A critical literature review & meta‐analysis. Cancer Epidemiology, 39, 923–938. 10.1016/j.canep.2015.09.013 26523982

[cre2153-bib-0035] Shield, K. D. , Ferlay, J. , Jemal, A. , Sankaranarayanan, R. , Chaturvedi, A. K. , Bray, F. , & Soerjomataram, I. (2017). The global incidence of lip, oral cavity, and pharyngeal cancers by subsite in 2012. CA: A Cancer Journal for Clinicians, 67, 51–64. 10.3322/caac.21384 28076666

[cre2153-bib-0036] Singhi, A. D. , & Westra, W. H. (2010). Comparison of human papillomavirus in situ hybridization and p16 immunohistochemistry in the detection of human papillomavirus‐associated head and neck cancer based on a prospective clinical experience. Cancer, 116, 2166–2173. 10.1002/cncr.25033 20186832

[cre2153-bib-0037] Smith, E. M. , Ritchie, J. M. , Summersgill, K. F. , Klussmann, J. P. , Lee, J. H. , Wang, D. , … Turek, L. P. (2004). Age, sexual behavior and human papillomavirus infection in oral cavity and oropharyngeal cancers. International Journal of Cancer, 108, 766–772. 10.1002/ijc.11633 14696105

[cre2153-bib-0039] Sobin, L. H. , Gospodarwicz, M. K. , & Wittekind, C. H. (2009). TNM classification of malignant tumours. Singapore: Wiley‐Blackwell Press.

[cre2153-bib-0040] Tuyns, A. J. , Estève, J. , Raymond, L. , Berrino, F. , Benhamou, E. , Blanchet, F. , … Zubiri, L. (1988). Cancer of the larynx/hypopharynx, tobacco and alcohol: IARC international case control study in Turin and Varese (Italy), Zaragoza and Navarra (Spain), Geneva (Switzerland) and Calvados (France). International Journal of Cancer, 41, 483–491. 10.1002/ijc.2910410403 3356483

[cre2153-bib-0041] WHO (2016). International statistical classification of diseases and related health problems ICD‐10. France: World Health Organization Press.

[cre2153-bib-0042] Witkiewicz, A. K. , Knudsen, K. E. , Dicker, A. P. , & Knudsen, E. S. (2011). The meaning of p16(ink4a) expression in tumors: Functional significance, clinical associations and future developments. Cell Cycle, 10, 2497–2503. 10.4161/cc.10.15.16776 21775818 PMC3685613

